# Effect of local anesthesia on pain scale and specimen adequacy in fine-needle aspiration biopsy of thyroid nodules for liquid-based cytology

**DOI:** 10.1038/s41598-022-23031-0

**Published:** 2022-11-02

**Authors:** Tsung-Lun Lee, Pei-Yin Wei, Shyh-Kuan Tai

**Affiliations:** 1 Department of Otolaryngology, National Yang Ming Chiao Tung University, Taipei, Taiwan; 2 Department of Otolaryngology, Taipei Veterans General Hospital, No 201, Sec 2, ShihPai Rd, Taipei, 11217 Taiwan; 3 Department of Otolaryngology, Taoyuan General Hospital, Ministry of Health and Welfare, Taoyuan, Taiwan

**Keywords:** Endocrinology, Health care, Medical research

## Abstract

Fine-needle aspiration biopsy (FNAB) is a routine diagnostic test for thyroid nodules. The use of local anesthesia (LA) before the procedure is still controversial. This prospective study aimed to evaluate the degree of pain and specimen adequacy in liquid-based cytology (LBC) for FNAB of thyroid nodules with and without LA. A total of 100 consecutive patients with thyroid nodules who underwent FNAB with and without LA between January and December 2020 were included. Patients who received LA had a significantly lower immediate pain scale score (P = 0.01). Multivariate analysis demonstrated that no use of LA (odds ratio [OR] = 3.48, 95% confidence interval [CI] = 1.50–8.10, P = 0.004) and lesion abutting the trachea (OR = 6.14, 95% CI = 1.56–24.12, P = 0.009) were independently and significantly associated with pain degree immediately after FNAB. A higher proportion of patients who previously underwent FNAB thought that LA was helpful and should be performed prior to FNAB. However, the use of LA did not improve the specimen adequacy (P = 0.075). The results showed that administration of LA with a proper technique before ultrasound-guided FNAB might reduce immediate pain after the procedure, and patients may experience more pain when the aspirated nodules abut the trachea.

## Introduction

Aspiration biopsy is a well-established technique that has been implemented in clinical practice over the past 150 years and is the recommended first line investigation for evaluation of masses, especially those in the head and neck area. Owing to its safety, accuracy, and simplicity, fine-needle aspiration biopsy (FNAB) of the thyroid gland remains the most cost-effective, accurate, and routinely used diagnostic tool in the initial evaluation of thyroid nodules^1–6^.

The factors that influence specimen adequacy during FNAB remain unclear. An ideal FNAB procedure should provide adequate specimens for cytological diagnosis, with minimal patient discomfort. The benefits and necessity for administering local anesthesia (LA) before FNAB are controversial and highly debated.

Compared to conventional smear (CS), liquid-based cytology (LBC) employs advanced concentration techniques to reduce the number of unsatisfactory and false positive results and has significantly improved cytodiagnostic accuracy^7,8^. Over time, LBC may replace the CS technique when performing thyroid FNAB, since LBC is useful for the cytological analysis of blood-contaminated aspirates; blood contamination of aspirates can occur due to the high vascularity of a thyroid nodule or the use of a large-caliber needle^7–9^.

The purpose of our study was to evaluate pain intensity levels in ultrasound-guided FNAB with or without LA in patients with thyroid nodules. Additionally, the influence of LA on the specimen adequacy of LBC was assessed.

## Results

### Patient characteristics

Between January and December 2020, 100 consecutive patients with thyroid nodules who underwent FNAB were enrolled. These patients had a median age of 53.0 (interquartile range, 42.0–62.0) years, and 67 (67.0%) were female. FNAB was performed with LA in 48 (48.0%) patients, and without LA in 52 (52.0%) patients. Patients with LA had a significantly greater proportion of previous FNAB experiences (70.8% vs. 34.6%, P < 0.001) than those without LA. There was no significant difference between the groups regarding demographics, procedural time, and nodule characteristics (Table 1).

**Table 1:** Demographics, procedural data, and characteristics of thyroid nodules in the patients undergoing FNAB with and without LA.

Variables	With LA (n = 48)	Without LA (n = 52)	P
Age (year)*	52.0 (36.5–61.0)	53.0 (46.0–63.5)	0.176
**Sex**			0.674
Female	31 (64.6)	36 (69.2)	
Male	17 (35.4)	16 (30.8)	
**Previous experience of FNAB**	34 (70.8)	18 (34.6)	< 0.001
**Procedural data**			
Needle size			0.665
18-gauge	15 (31.3)	14 (26.9)	
21- and 23-guage	33 (68.8)	38 (73.1)	
Number of needle passes*	6 (2–9.5)	9 (3–12)	0.121
Procedure time (second)*	60.5 (36.0–96.0)	61.0 (41.5–109.0)	0.649
**Nodule characteristics**			
Size of nodule (mm) *	19.0 (12.0–29.5)	20.0 (13.0–23.5)	0.988
Depth of nodule*			
Depth 1 (mm)	23.0 (19.0–30.0)	22.0 (18.0–31.0)	0.764
Depth 2 (mm)	7.5 (5.0–11.0)	8.0 (6.0–11.5)	0.313
Cystic lesion	18 (37.5)	18 (34.6)	0.836
Lesion against trachea	6 (12.5)	4 (7.7)	0.514
ATA sonographic risk stratification			0.888
Benign	2 (4.2)	2 (3.8)	
Very low risk	17 (35.4)	15 (28.8)	
Low risk	13 (27.1)	19 (36.5)	
Intermediate	4 (8.3)	5 (9.6)	
High risk	12 (25)	11 (21.2)	

*Data are presented as counts (percentages) or medians (interquartile ranges).

FNAB, fine-needle aspiration biopsy; LA, local anesthesia; ATA, American Thyroid Association; NA, not available.

### Results of pain assessment

As presented in Table 2, the distribution of pain scores immediately after FNAB was significantly different between patients with and without LA (P = 0.010). The group with LA use had a significantly lower level of pain intensity immediately after the procedure. At 5 min after the procedures, the difference in the degree of pain became insignificant between the two groups. Regarding the subjective experience of pain, the patients with LA reported more pain at the superficial site (54.8% vs. 29.4%, P = 0.03) and less stabbing pain (58.1% vs. 67.7%, P = 0.42) than those without LA. Among patients with prior FNAB experience, significantly more patients in the LA group considered this FNAB experience to be less painful (79.4% vs. 16.7%, P < 0.001) compared to those without LA. Meanwhile, patients with a previous experience in the LA group were significantly more likely to report that LA is necessary prior to FNAB compared to those without LA (79.4% vs. 5.6%, P < 0.001).

**Table 2:** Pain characteristics in the patients undergoing FNAB with and without LA.

Variables	With LA (n = 48)	Without LA (n = 52)	P
**Pain score immediately after FNAB**			0.010
0	8 (16.7)	3 (5.8)	
2	29 (60.4)	26 (50)	
4	10 (20.8)	16 (30.8)	
6	0 (0)	7 (13.5)	
8	1 (2.1)	0 (0)	
10	0 (0)	0 (0)	
**Pain score 5 min after FNAB**			0.356
0	45 (93.8)	46 (88.5)	
2	1 (2.1)	5 (9.6)	
4	2 (4.2)	1 (1.9)	
6	0 (0)	0 (0)	
8	0 (0)	0 (0)	
10	0 (0)	0 (0)	
**Subjects with experience of FNAB (n = 52)**			
Comparison with previous exam			< 0.001
Greater pain	0 (0)	4 (22.2)	
Similar pain	7 (20.6)	11 (61.1)	
Less pain	27 (79.4)	3 (16.7)	
Need for LA	27 (79.4)	1 (5.6)	< 0.001

Data were presented as count (percentage).

FNAB, fine-needle aspiration biopsy; LA, local anesthesia.

### Cytological results

The non-diagnostic sample rate was 13% in our study (n = 100). Although the rate of specimen adequacy was higher in FNAB performed under LA, the result was not statistically significant (93.8% vs. 80.8%, P = 0.075) (Table 3). There was no significant difference in the Bethesda System for Reporting Thyroid Cytopathology between the two groups (P = 0.462).

**Table 3:** Cytological results of patients undergoing FNAB with and without LA.

	With LA (n = 48)	Without LA (n = 52)	P
**Specimen adequacy**			0.075
Inadequate	3 (6.3)	10 (19.2)	
Adequate	45 (93.8)	42 (80.8)	
**Bethesda classification**			0.462
I	3 (6.3)	9 (17.3)	
II	39 (81.3)	37 (71.2)	
III	1 (2.1)	1 (1.9)	
IV	0 (0)	0 (0)	
V	2 (4.2)	1 (1.9)	
VI	3 (6.3)	4 (7.7)	

Data were presented as count (percentage).

FNAB, fine-needle aspiration biopsy; LA, local anesthesia.

### Association of local anesthesia with pain

Ordinal logistic regression was used to evaluate factors associated with immediate post-procedure pain intensity (Table 4). For the univariate analysis, no LA (odds ratio [OR] = 2.94, 95% confidence interval [CI] = 1.33–6.51, P = 0.008) or lesion against trachea (OR = 6.48, 95% CI = 1.83–23.01, P = 0.004) were significantly related to immediate pain scale. The Wong-Baker FACES Pain Rating Scale immediately after the procedure was not associated with needle size (the average scores and standard deviations for 18 gauge needle and 21–23 gauge needles were 2.41 ± 1.24 vs. 2.73 ± 1.73), number of needle passes, depth of the nodule and American Thyroid Association (ATA) sonographic risk stratification. Six variables with P < 0.2 in the univariate analysis were included in the multivariate analysis, including procedural time, administration of LA, nodule size, lesion against the trachea, and specimen adequacy. The results of multivariate analysis revealed that no LA (OR = 3.48, 95% CI = 1.50–8.10, P = 0.004) and lesion against the trachea (OR = 6.14, 95% CI = 1.56–24.12, P = 0.009) were independently and significantly associated with the degree of pain immediately after FNAB (Table 4).

**Table 4:** Ordinal logistic regression for the associated factors of degree of immediate pain after FNAB.

Variables	Univariate		Multivariate	
	OR (95% CI)	P	OR (95% CI)	P
**Age (year)**	0.99 (0.97–1.02)	0.680		
**Sex**				
Female	Reference			
Male	1.47 (0.66–3.28)	0.342		
**Experience of FNAB**				
No	Reference			
Yes	1.06 (0.50–2.25)	0.881		
**Needle size**				
18-gauge	Reference			
21- and 23-guage	1.26 (0.55–2.91)	0.581		
**Number of needle passes**	1.02 (0.95–1.09)	0.640		
**Procedure time (second)**	1.00 (1.00–1.01)	0.114	1.00 (1.00–1.01)	0.478
**Use of LA**				
LA	Reference		Reference	
No LA	2.94 (1.33–6.51)	0.008	3.48 (1.50–8.10)	0.004
**Size of nodule (mm)**	0.97 (0.94–1.01)	0.142	0.99 (0.95–1.03)	0.530
**Depth of nodule**				
Depth 1 (mm)	0.98 (0.94–1.03)	0.483		
Depth 2 (mm)	1.03 (0.94–1.12)	0.545		
**Cystic lesion**				
No	Reference			
Yes	0.97 (0.44–2.12)	0.935		
**Lesion against trachea**				
No	Reference		Reference	
Yes	6.48 (1.83–23.01)	0.004	6.14 (1.56–24.12)	0.009
**ATA sonographic risk stratification**				
Benign	Reference			
Very low risk	0.61 (0.08–4.64)	0.634		
Low risk	1.32 (0.18–9.93)	0.785		
Intermediate	1.35 (0.14–13.13)	0.796		
High risk	1.85 (0.24–14.42)	0.558		
**Specimen adequacy**				
Inadequate	Reference		Reference	
Adequate	0.42 (0.14–1.25)	0.120	0.75 (0.23–2.45)	0.635

FNAB, fine-needle aspiration biopsy; LA, local anesthesia; ATA, American Thyroid Association; NA, not available; OR, odds ratio; CI, confidence interval.

## Discussion

FNAB is regarded as a simple, safe, and cost-effective tool for evaluating thyroid nodules^7^. One of the main reasons for patient dissatisfaction with service quality is pain during the procedure^10^. To this end, LA has been used in FNAB of thyroid nodules to reduce pain and improve patient satisfaction with the quality of services^11,12^. Administration of LA may facilitate multiple and more accurate aspirates without more pain. However, the requirement and indications for LA remain controversial. Several researchers^13–15^ suggested that FNAB should be performed without LA when only one needle puncture is used. However, Gursoy et al.^16^ confirmed that a topical anesthetic cream could provide effective and non-invasive analgesia during FNAB of thyroid nodules. In addition, Liao et al.^17^ reported that the use of LA was associated with not only lower immediate pain scales but also higher specimen adequacy.

To our best knowledge, the present study is the first prospective trial to compare the pain scale and specimen adequacy between groups receiving FNAB of thyroid nodules with or without LA using LBC. We found no significant differences in age; sex; size, depth, and composition of thyroid nodules; needle size; number of needle passes; procedures; ATA nodule sonographic patterns; and risk of malignancy between the subjects receiving FNAB with or without LA. However, patients with LA had a higher rate of experience with FNAB (70.8% vs. 34.6%, P < 0.001). Administration of LA before FNAB was related to a significantly lower immediate pain scale score (P = 0.010), but this was not sustained at 5 min after the procedure (P = 0.356).

Several factors, including the use of LA^16–18^, characteristics of thyroid nodules^17,19^, and number of biopsied thyroid nodules^20^, have been reported to be associated with pain score after FNAB. However, the degree of pain after FNAB was not related to needle size^21–23^. In our study, we found that the absence of LA and the presence of a thyroid nodule against the trachea were independently and significantly associated with greater pain intensity after adjusting for multiple factors. Furthermore, among the patients with previous experience of FNAB, a higher proportion of the subjects who received LA felt less pain during this procedure and thought LA use is necessary compared with those without LA.

There are some differences between the results obtained in our study and those of previous reports and therefore our results should be interpreted cautiously. In our study, smaller caliber needles (27-gauge) were used for LA. A well-designed randomized controlled trial^24^ showed an inverse relationship between needle diameter and pain. In addition, we used 1% lidocaine instead of 2% lidocaine because 1% lidocaine or buffered lidocaine with bicarbonate may cause less discomfort than 2% lidocaine^25^. Finally, we waited for 10 s after subdermal injection of LA, and did not proceed with further administration of LA until the patient reported that the needle-puncture pain had disappeared. This pause allowed the local anesthetic to alleviate the pain caused by the needle piercing into the skin. If properly performed, this technique allows the subject to not feel any pain after the first puncture^26^.

It is worth noting that the subjects in our study had more intense pain when their thyroid nodules abutted the trachea. This phenomenon has not been previously discussed. The majority (~ 75%) of afferent activities from sensory terminals located in the airway, including the larynx and trachea, are mediated by non-myelinated C-fibers^27^. This may account for the pain sensation when the needle approached the trachea during FNAB. However, further studies should be conducted to clarify whether LA should be recommended in patients with lesions touching the trachea given that they are more likely to experience more pain during the procedure.

LBC has been used increasingly for thyroid FNAB because of its advantages over CS, including tolerance of bloody aspirates, less operator dependency on FNA, and the applicability of immunohistochemistry and molecular biology^7,8^. However, thyroid cytomorphology is more clearly seen on CS than in LBC^28^, and both of them are helpful, depending on the situation. In FNAB of thyroid nodules, the relationship between needle size and specimen adequacy in LBC is different from that in CS. No significant difference was found in the cytological adequacy between the different needle gauges using LBC^22,23^. To the best of our knowledge, no previous studies have assessed the effect of administration of LA on the specimen adequacy in LBC. Liao et al.^17^ reported that the use of LA was related to higher specimen adequacy in CS. However, our results demonstrated that regardless of whether the patients received LA or not, the rate of harvested cytological adequacy was not significantly different in LBC. The reason for this discrepancy is unclear, especially as LBC overcomes the drawback of bloody aspirate contamination that hinders visual analysis in CSs^29^. Further studies are required to clarify this matter.

The present study has several limitations. First, the patients with and without LA were not assigned randomly, and administration of LA was not performed in a blind method. No LA may affect the patient’s psychological expectation of pain, which has an influence on the pain scores. Second, our study included a relatively small number of participants, and there were far fewer male participants than female participants. Finally, the US-FNAB technique included only one sample with a single puncture. Accordingly, this study result should not be applied to FNAB with two or more samplings.

In conclusion, this study demonstrated that LA administration before FNAB using a proper technique reduced immediate pain after the procedure, and patients with thyroid nodules abutting the trachea may experience more pain during FNAB. Among the patients with previous experience of FNAB, a higher proportion of the subjects who received LA felt less pain during the procedure and thought LA use was necessary compared with those without LA.

## Material and methods

### Patient selection

This prospective study was conducted at Taipei Veterans General Hospital, a tertiary referral medical center, from January 2020 to December 2020. The study protocol was approved by the Institutional Review Board of the Taipei Veterans General Hospital (No. 2019-02-023A). All methods were performed in accordance with the relevant guidelines and regulations. Adult patients (aged > 20 years) who underwent ultrasound-guided FNAB for thyroid nodules were enrolled. Patients who underwent FNAB for assessment of cervical lymph nodes or other head and neck masses, had painful thyroid lesions, or had a history of allergy to LA were excluded. Written informed consent was obtained before the FNAB procedure from individual participants included in the study. All patient-identifiable data were anonymized.

### Ultrasound-guided FNAB

Ultrasound-guided FNAB was performed by an experienced head and neck surgeon (L.-T.L.; has more than 5 years’ experience performing ultrasound-guided FNAB in over 1000 cases) using an Aloca Arietta 70 with a specialty transducer L55 high-resolution 13.0-MHz to 5.0-MHz 50 mm width real-time linear-array transducer. Patients were examined in the supine position with an extended neck for better exposure. The aspirated nodule was determined according to the most suspicious nodule on US visualization. The overlying skin area was sterilized with 75% alcohol solution.

Ultrasound-guided FNAB was performed using a freehand long-axis approach and positioning the linear array probe parallel to the needle for guidance. The needle was placed in the thyroid nodule under ultrasound guidance. A 21-gauge or 23-gauge needle attached to a 3-mL syringe was used for puncture, and an 18-gauge needle was applied for cystic or predominantly cystic (fluid component > 50%) thyroid nodules to drain the liquid content as much as possible. Only one needle puncture site was performed despite multiple needle passes, and the number of needle passes depended on the clinical suspicion.

Procedural data including needle size, number of needle passes during the procedure, and procedure time were collected. The characteristics of the nodules were also evaluated including the largest diameter of the thyroid nodule, depth of the nodule, whether the lesion abuts the trachea, presence of cystic lesions, and ATA nodule sonographic patterns and risk of malignancy^30^. The measures for the depth of the nodule included the distances between the skin and the superficial surface of the thyroid nodule (depth 1) and between the skin and the deepest surface of the thyroid nodule (depth 2).

FNAB with or without LA was performed alternatively in consecutive patients. Briefly, the first patient underwent FNAB without LA and the second patient underwent FNAB with LA. However, if the patient refused the arrangement, the next subject still followed the sequence. Therefore, less than half of the study patients received LA. First, approximately 0.2 mL of 1% lidocaine hydrochloride solution was injected subdermally. Following a 10 s pause, additional LA was injected after the patient reported that the needle-puncture pain had disappeared. The residual LA (a total of 1 mL) was injected into the soft tissue between the thyroid capsule and the skin overlying the target nodule with a 27-gauge needle attached to a 3-mL syringe.

### Liquid-based cytology and cytological analysis

After centrifugation of the CytoLyt™ solution containing the aspirates, the obtained pellet was transferred to PreservCyt™ (Hologic-Cytyc Co.) solution and allowed to sit for 15 min. A single Papanicolaou-stained slide was prepared for each case using ThinPrep 5000 (Hologic-Cytyc Co.).

All ThinPrep slides were reviewed by two cytopathologists who specialized in cytological analysis of thyroid FNAB specimens and were blinded to the clinical data. Specimen adequacy was assessed using the Bethesda System for Reporting Thyroid^31^. The criteria for specimen adequacy consisted of at least six groups of follicular cells, and each group contained at least 10 cells and adequate cells for cytological analysis. Specimens that contained only abundant colloid or numerous inflammatory cells were considered inadequate. Specimens that consisted only of cystic contents were considered inadequate.

### Pain assessment

The Wong-Baker FACES Pain Rating Scale^29,32^ was used to rate the degree of pain at two time points: immediately and 5 min after the completion of ultrasound-guided FNAB. The scale provides a six-item ordinal face scale. An incremental value of 2 is added for each face as the scale moves from to 0–10, ranging from a happy face at a pain score of 0 to a crying face at 10 (0 = no pain, 10 = worst pain). The patients were also interviewed after the procedure regarding the subjective experience of pain location (e.g., superficial, deep) and types of pain (e.g., stabbing, blunt, dull, pressure pain). Subjects who had previously undergone FNAB were also requested to compare the degree of pain between the two experiences (greater, similar, or less pain) and provide their opinion on the need for LA.

### Statistical analysis

Categorical or ordinal variables were presented as counts (percentages) and continuous variables as medians (interquartile ranges). Comparisons between patients with and without LA were performed using Fisher’s exact test for categorical or ordinal variables, and the Mann–Whitney U test for continuous variables. Ordinal logistic regression was used to evaluate factors associated with pain degree immediately after FNAB. Variables with P < 0.2 in the univariate analysis were included in the multivariate model. Results of ordinal logistic regression analysis were presented as odds ratios (ORs) with 95% confidence intervals (CIs). Statistical significance was set at P < 0.05. All analyses were conducted using IBM SPSS Statistics for Windows version 26.0 (IBM Corp., Armonk, NY, USA).
